# Does environmental stress affect cortisol biodistribution in freshwater mussels?

**DOI:** 10.1093/conphys/coz101

**Published:** 2019-12-08

**Authors:** A Ronja D Binder, Michael W Pfaffl, Felix Hiltwein, Juergen Geist, Sebastian Beggel

**Affiliations:** 1 Animal Physiology and Immunology, School of Life Sciences Weihenstephan, Technical University of Munich, Weihenstephaner Berg 3, Freising-Weihenstephan, D-85354, Germany; 2 Independent Scholar; 3 Aquatic Systems Biology Unit, School of Life Sciences Weihenstephan, Technical University of Munich, Mühlenweg 22, Freising-Weihenstephan, D-85354, Germany

**Keywords:** *Anodonta anatina*, bioindication, cortisol ELISA, steroid hormones, invertebrates, bivalves

## Abstract

As of today, regulation and physiological purpose of steroid hormones in invertebrates such as mussels are not completely understood. Many studies were able to show their presence, but their origin and genesis are not clear. Nevertheless, knowledge about changes in steroid hormone biodistribution in reaction to treatments could improve our understanding of their physiological functions in these species. Cortisol is a corticosteroid, which is frequently used as a stress biomarker in vertebrates, like fish or higher organisms. The aim of the study was to optimize cortisol extraction from various tissues of mussels, to develop a quantitative ELISA test system, and to study changes in biodistribution of cortisol in reaction to negative and positive stimulation treatments. As model organism, we used *Anodonta anatina,* a widespread freshwater mussel species native to Europe. We quantified cortisol concentrations in hepatopancreas, mantle, gills, gonads and the foot muscle. Tissue-specific reactions to environmental influences, simulated with the chemical stressors copper (II) chloride and sodium chloride, were assessed. During the 24-hours treatment, we additionally observed changes in cortisol regulation in response to feeding activity of the mussels. Besides, we found highly significant variations in the biodistribution of cortisol in different tissues, with a peak in the hepatopancreas. Whole body cortisol did not increase in the treated groups. However, balancing of all measured tissues showed redistribution of more than 10% of total body cortisol from the hepatopancreas to all other tissues during copper (II) chloride stressor treatment, but also when mussels ingested feed, compared to the non-fed control group. No redistribution was observed during sodium chloride treatment. We conclude that there can be a redistribution of cortisol in mussels, depending on external influences.

## Teaser

Lay summaries:

Since little is known about invertebrate steroid hormones, we established a cortisol extraction and quantification protocol via ELISA in mussels, in the model organism *Anodonta anatina*. Additionally, we quantified cortisol biodistribution and redistribution in five different tissues after treatment with either copper (II) chloride, sodium chloride or algae feed.

## Introduction

Freshwater unionoid mussels rank among the most endangered taxonomic groups worldwide (Lydeard *et al.*, 2004; Dudgeon *et al.*, 2006; Lopes-Lima *et al.*, 2017) and are considered target species of conservation (Geist, 2010, 2015). Mussels play a key role in ecosystem processes such as nutrient cycling, particle clearance and biodeposition (Lummer *et al.*, 2016; Vaughn, 2018). They are important for the provision of lotic and lentic ecosystem services (Strayer *et al.*, 2004; Geist, 2011; Cardinale *et al.*, 2012; Vaughn, 2018). Anthropogenic activities such as habitat alterations, introduction of invasive species, as well as point source and diffuse pollution are the main reasons for the global decline of freshwater mussel biodiversity and abundances. Disturbances of ecosystem functioning and health are the consequences (Cardinale *et al.*, 2012). As sessile filter feeders, freshwater mussels are in direct contact with any kind of water-soluble or particulate substance in their surrounding media (Tankersley and Dimock Jr, 1993; Lopes-Lima *et al.*, 2017). This makes them specifically vulnerable for changes in water quality caused, for example, by chemical contaminants. Some chemicals (Roméo *et al.*, 2005), but also biological pathogens (Palos Ladeiro *et al.*, 2014), induce reactions in the mussels, like changes in their filtering performance (Coughlan, 1969; Hartmann *et al.*, 2016; Lummer *et al.*, 2016; Beggel *et al.*, 2017), and some accumulate in the organisms. Regarding pollution control in rivers or lakes, a reliable biomonitoring system is indispensable (Viarengo and Canesi, 1991; Sheehan *et al.*, 1995; Minguez *et al.*, 2011). For an optimal use of bioindicators like mussels, it is necessary to quantify the consequences of environmental stressors under controlled conditions. A common approach for quantifying stress would be the measurement of different kinds of biomarkers in body fluid or tissue following defined exposure scenarios. A range of physiological stress biomarkers have been identified in marine bivalves (Liu *et al.*, 2004; Li *et al.*, 2007); however, information on freshwater mussels remains scarce (Fritts *et al.*, 2015). In other, mainly vertebrate, species, a frequently used biomarker for environmental effects and stress-induced effects is the corticosteroid cortisol (Hellhammer *et al.*, 2009; Yeh *et al.*, 2013; Gong *et al.*, 2015). The regulation of this glucocorticoid is well understood for vertebrates such as mammals and fish, but little is known about its role in invertebrates. In mammals, birds and some other vertebrate species, cortisol is produced in the *zona fasciculata* in the adrenal cortex. Cortisol is released in increased concentrations when a stressor is interfering with the organism (Hellhammer *et al.*, 2009; Etwel *et al.*, 2014; Gong *et al.*, 2015; Nohara *et al.*, 2016). In various species, a range of not purely stress-related functions of cortisol have also been described (Krieger, 1979; Santiago *et al.*, 1996; Boland *et al.*, 2004). In mussels, no structure such as an adrenal cortex is known. Nonetheless, the presence of cortisol (Ottaviani *et al.*, 1998) in mollusks has been described, although there is still an ongoing and controversial discussion about the validity of steroid hormone presence in invertebrates (Lafont and Mathieu, 2007; Fernandes *et al.*, 2011). On the one hand, steroid hormones were detected in invertebrates with different methods (Lupo Di Prisco *et al.*, 1973; Jong-Brink *et al.*, 1981; Le Guellec *et al.*, 1987; Reis-Henriques *et al.*, 1990), and there are indications for synthetic pathways of steroid hormones in invertebrates (Lupo Di Prisco *et al.*, 1973; Lupo di Prisco and Dessi’ Fulgheri, 1975; Le Guellec *et al.*, 1987). On the other hand, it is assumed that steroid hormones are environmentally accumulated substances, which do not fulfil hormone roles in invertebrates (Lafont and Mathieu, 2007; Fernandes *et al.*, 2011). Environmental accumulation could be explained by non-specific, lipophilic binding in tissues rich in fat. Nevertheless, regardless of the source of steroid hormones in invertebrates, many studies were able to show correlations between the level of steroid hormones and different internal or external influences on invertebrates (Gooding and LeBlanc, 2005; Riva *et al.*, 2010; Lazzara *et al.*, 2012). In mollusks in particular, a correlation between different kinds of sexual steroid hormones and reproductive processes is assumed (Reis-Henriques *et al.*, 1990; Gooding and LeBlanc, 2005; Omran, 2012). While environmental steroid hormones would be absorbed by filter feeders easily, an uptake would be less likely for non-filter feeders. Despite being non-filter feeder mollusks, steroid hormones were found in aquatic snails (Gooding and LeBlanc, 2005; Omran, 2012) and terrestrial snails (Le Guellec *et al.*, 1987). For cortisol, a correlation with stress or another internal or external influence has, to our current knowledge, not been demonstrated for freshwater mussels so far. Enhanced background knowledge about the biodistribution of cortisol in mussels would allow a broader understanding of the function of steroid hormones in invertebrates in general and of cortisol in mussels in particular. If any changes in the cortisol level during a model treatment are measureable, it could improve the application of cortisol as a stress biomarker within these species as well, regardless of cortisol genesis. This would thus improve the value of mussels as environmental bioindicator organisms (Hooper *et al.*, 2007).

To determine the biodistribution of cortisol in mussels, we extracted cortisol from five different tissues of the freshwater mussel *Anodonta anatina* (mantle, gills, hepatopancreas, foot muscle and gonads). Afterwards, we quantified cortisol using an ELISA. For this reason, we established a cortisol extraction and ELISA procedure for mussels. In addition to the cortisol measurement protocol in mussels, we exemplarily monitored possible effects of environmental influences on cortisol level in select tissues (pg cortisol per gramme of tissue) and total body cortisol in freshwater mussels. We modelled these influences with two chemical distress treatments: copper (II) chloride (CuCl_2_) and sodium chloride (NaCl). In the mussels’ natural habitat, possible sources for NaCl can be road meltwater input or salt mining (Beggel and Geist, 2015). For copper, excessive application of copper-based fungicides or mineral fertilizers, which can also contain copper, can result in contamination of soils and surface water bodies. Both salts are known to act as toxins on mussels at certain concentrations and are therefore considered suitable model substances to induce chemical stress (Hartmann *et al.*, 2016; Wang *et al.*, 2007a, 2007b). Since there are diverse influences affecting mussels that are still relatively unexplored and since studies exist, which are hypothesizing that eustress could cause effects similar to distress (Koolhaas *et al.*, 2011; Buwalda *et al.*, 2012; Villalba and Manteca, 2019), we decided to treat the animals with a stimulation model in addition to the distress treatments. As the stimulant, we chose feeding of the mussels.

## Material and methods

### Characterization and husbandry conditions of *A. anatina*

*A. anatina* were obtained from a commercial aquaculture (KoiCompetence, Germany). Acclimatization phase after arrival was for at least 7 days. During this time, mussels were kept under flow-through conditions (~10% water exchange per hour) at the following water parameters: mean ± SD; temperature 12.3 ± 0.5°C; dissolved oxygen (DO) 8.9 ± 1.2 mg L^−1^; and electric conductivity (EC, at 25°C) 638.70 ± 81.97 μS cm^−1^. Continuously oxygenated tap water was used. The ionic composition of the water is shown in the supplementary (Table S1, water-chemistry parameters). Light conditions were 12:12 h dark–light during acclimatization period. Every mussel was weighted, and its maximum length and width was measured. Living body weight (wet weight) averaged 40.32 ± 11.38 g (mean ± SD), the length 75.85 ± 7.26 mm, the width 41.7 ± 3.85 mm and the height 21.85 ± 2.88 mm. Mussels were individually marked with a waterproof marker for identification. During the acclimatization period, the mussels were fed with algae (Instant Algae; Nanno 3600, CCMP525, *Nannochloropsis* sp., algae TOC content 25.13 mg/L, California, USA), ~15 ml per 60 mussels every second day diluted 1:10 (v/v). Species identity was confirmed genetically (Zieritz *et al.*, 2012).

### Experimental setup

For the experiment, the mussels were randomly assigned to three groups of 20 individuals each, a control group and two treatment groups that were either exposed to CuCl_2_ or NaCl for 24 hours. Before the experiment started, mussels were fasted for 3 days. We fed the mussels, 1 hour after the experiment started, with 5 ml of algae per mussel (Instant Algae; Nanno 3600, CCMP525, *Nannochloropsis sp*., CA, USA, algae TOC content 25.13 mg/L*),* diluted 1:10 (v/v). Therefore, we further subdivided all distress treatment groups into one part without feed and one part provided with feed. This means that six different types of treatments were created: standard treatment, standard treatment with feed, copper (II) chloride treatment, copper (II) chloride treatment with feed, NaCl treatment and NaCl treatment with feed. Filtering activity of mussels was assessed during and after the experiment (Table 2). Basis for this was measured filtration rate and filling of gastrointestinal tract of the animals (Table 2). No individual mussel of the distress treatments showed filtering activity. We therefore clustered the six types of treatments into four groups and defined them as ‘control group that had no positive feed intake’ (CNF, *n* = 10), ‘control group that had positive feed intake’ (CF, *n* = 10), ‘copper (II) chloride-treated group’ (CuCl_2_, *n* = 20) and sodium chloride-treated group (NaCl, *n* = 20). Every individual was kept in a separated, 1-L glass beaker with tap water. Water parameters were measured in every single glass beaker. Water temperature was adjusted to 11.38 ± 0.41 °C (mean ± SD) and dissolved oxygen averaged 8.13 ± 0.09 mg L^−1^. The electric conductivity averaged 638.70 ± 81.97 μS cm^−1^ for the control group, 664.80 ± 80.36 μS cm^−1^ for the CuCl_2_-treated group and 18.00 ± 0.44 mS cm^−1^ for the NaCl-treated group. The pH averaged 8.56 ± 0.04 for the control group, 8.52 ± 0.04 for the CuCl_2_-treated group and 8.57 ± 0.06 for the NaCl-treated group. The measurements were done with the following equipment (all WTW, Germany): conductivity, Cond 3110; oxygenation, oxygen probe Oximeter Oxi 320; and pH, pH meter 340i. An air pump oxygenated every single beaker. For temperature equalization, the glass beakers were put into a flume with water. For the treatments, either CuCl_2_ (Carl Roth GmbH, Germany) or NaCl (crystal, J.T.BAKER, Netherlands) was added. The nominal concentration of CuCl_2_ and NaCl was based on known toxicity data for freshwater mussels (Rao and Khan, 2000; Wang *et al.*, 2007a, 2007b, 2018b, 2018a; Negri *et al.*, 2013; Davis *et al.*, 2018; Lettieri *et al.*, 2019). For CuCl_2_, stock solutions were prepared with a concentration of 300 mg L^−1^ and transferred to the exposure vessels to yield the final test concentration of 0.25 mg L^−1^. Afterwards, test concentration of copper was verified using Spectroquant colorimetric test kit (Merck, Germany). It averaged 0.24 ± 0.03 CuCl_2_ mg L^−1^ (*n* = 20, mean ± SD). For the NaCl treatment, 10 g L^−1^ NaCl were weighted in. Additionally, the electric conductivity was identified in every glass beaker, and the NaCl concentration was adjusted, based on a standard curve. The measured NaCl concentration averaged 10.26 g L^−1^ (coefficient of determination [*R*^2^] = 0.9952). The glass beakers rested for several hours after the salt application to allow proper mixing of the test solution. Because of the caging in a glass beaker and without substrate during the experiment, an adsorption of the salts to the used equipment was not to be expected. Further water-chemistry parameters (potassium, calcium, magnesium, iron, manganese and water hardness) were measured and are listed in the supplementary (Table S1, water-chemistry parameters). Parameters for sodium and chloride are also listed in the supplements, with the exception of the treated groups in which sodium (Na^+^) and chloride (Cl^−^) were increased. The concentration for the NaCl treatment averaged 4.04 (sodium) and 6.22 g L^−1^ (chloride). For the CuCl_2_ treatment, the chloride (CL^−^) concentration only increased marginally (increase of 0.38 g L^-1^, mean ± SD).

### Filtering activity

Filtering activity was evaluated in three different ways:

- During the treatment, visible filtering of the mussels was observed, i.e. the opening of the mussels and a visible siphon was recorded.
- Before dissection, the caging water was assessed, based on water turbidity.
- During the dissection, the content of the gastrointestinal tract of each mussel was noted.

### Dissection and homogenization

To avoid the contamination of samples with human-derived biological particles or sweat during handling of the animals and during cortisol extraction and measurement, gloves were used, hair was tied back, and skin-covering cloth was worn. To minimize external influence on results, every step of the following protocol was done with animals of different treatments (i.e. test groups) at the same time. Negative controls were included. After 24 hours of treatment, the mussels were taken from their glass beakers and immediately sacrificed with a sharp knife by cutting through the hinge to puncture the heart. Afterwards, the mussel was opened carefully while dissociating the mantle from the shell. Every mussel was dissected into seven macroscopically visible sections: mantle, gills, foot, kidney, hepatopancreas (HP) (in combination with the stomach), heart and gonads (in combination with most of the intestine). All tissues were weighted, directly put on ice, and immediately frozen at −20°C. After a few hours, they were transferred to −80°C and stored at this temperature until further analysis. After removing the remaining soft parts, the empty shells were weighted. To avoid possible stress for the animals, ‘whole mussel wet weight’ was measured only before the treatments and not directly before dissection.

Frozen tissues (mantle, gills, foot, hepatopancreas and gonads) were completely homogenized with a homogenizer (Art-Miccra-D-8; 1–6 min, depending on the tissue type and mass) and placed on ice. Between every change of sample, all parts of the homogenizer, which had been exposed to the homogenate, were cleaned with three washing steps (bi-distilled water—ethanol purified—bi-distilled water). After every wash step, drying with a clean paper towel was done. Homogenates were either frozen at −80°C or used for cortisol extraction directly.

### Cortisol extraction

Cortisol extraction was done with ethyl acetate (≥99.5%, AnalaR Normapur ACS, Reag. Ph. Eur. per analysis, VWR, Germany) and a 30% tert-butyl methyl ether (MTBE, ≥99.5%, pur, AppliChem, Germany) and 70% petroleum ether (40–60 °C, ≥99.5%, residue analysis grade, AppliChem, Germany) mixture (v/v). Due to safety aspects, the formerly used extraction of cortisol by di-ethyl ether was replaced with ethyl acetate and a 30% MTBE and 70% petroleum ether mixture. The substances were handled with the required care. All steps, which included the usage of either the ether mixture or ethyl acetate, were performed under a fume hood and with the appropriate personal protection. During the complete extraction procedure, only glassware, like glass pipettes and glass tubes, was used. For cortisol extraction, a certain amount of every homogenate was aliquoted. The mass of the samples ranged from 0.20 up to 0.64 g. Depending on the mass, an appropriate volume of ethyl acetate was added to the samples at room temperature (0.2–0.24 g, 2 mL; 0.25–0.34 g, 3 mL; 0.35–0.44 g, 4 mL; 0.45–0.54 g, 5 mL; 0.55–0.64 g, 6 mL). Samples were vortexed thoroughly for 30 seconds and incubated for 15 minutes at room temperature. Then they were frozen at −60°C for 1 hour. The samples were thawed at room temperature until all ice crystals in the liquid phase had vanished. Then an admeasured volume of the liquid phase was taken (1.5 up to 5.5 mL) and transferred into a clean test tube. The test tubes with the liquid were centrifuged in a vacuum concentrator/centrifugal evaporator (Jouan RC 10.22) combined with a vacuum pump (Vacuubrand) with 1200 rpm in the ‘no-heating’ modus of the centrifuge, until only a pellet remained, for 1–3 hours (time depended on the filling of the centrifuge). After centrifugation, the pellets were resuspended in a 200-μL ether mixture, composed of 70% petroleum ether and 30% MTBE. In addition, the whole test tube was washed with the mixture, so that cortisol residues could be washed off the test tube. The samples were dried overnight. To the pellet, 200-μL ELISA buffer and 10 μL of the ether mixture were added. The test tube was vortexed for 20 seconds or until the pellet was dissolved. Then, it was dried at room temperature until the ether had completely evaporated and only the ELISA buffer with the cortisol remained.

### Establishment of the cortisol ELISA

As most ELISA kits are not designed for assessing mussel extracts, a competitive enzyme-linked immunosorbent assay (ELISA) and workflow were developed. Only freshly isolated cortisol extracts were used for the measurement. Buffers were based on publications of Meyer (1989), Prakash *et al.* (1988)) and Yadav *et al.* (2013). Detailed information, about their partially modified compositions, can be found in the supplementary material. Polystyrene plates (Nunc MaxiSorp, 96 well, MicroWell, Denmark) were coated with a goat anti-rabbit antibody (purified as established by Meyer, 1989), with the use of the method described by Prakash *et al.* (1988), blocked with assay buffer and frozen at −20°C with a small residuum of assay buffer, for storage. Before use, the plates were thawed at room temperature and rinsed with 280 μL of washing buffer two times. Hence, the next steps were performed at 4°C. 100 μL cortisol antibody (antibody against antigen, 4-pregnen-11b 17a,21-triol-3,20dior-21-HS-BSA in rabbit serum, immunized as established by Meyer (1989); dilution in assay buffer, 1:90 000) was added and incubated at 4°C for 10 minutes. Then, 20 μL of a sample were added and also immediately 100 μL of cortisol-glucuronide horseradish peroxidase (HRP) complex (Meyer, 1989) (dilution in assay buffer: 1:12 000). The mixture incubated at 4°C for 16 hours on a shaker in the dark. The next steps were performed at room temperature. Plates were rinsed four times with 280 μL of washing buffer; reaction substrate A was pre-heated and mixed with reaction substrate B, so that 27°C were reached. 150 μL of the pre-heated reaction substrate mix were loaded and incubated for 40 minutes on a shaker at room temperature, in the dark. The reaction was stopped by adding 50 μL of a two molar sulfuric acid (AppliChem, Germany) and evaluated with an absorbance microplate reader (TECAN sunrise; Magellan software). For each ELISA plate, a new standard curve, in the range of 0.5 pg cortisol to 125 pg cortisol per well, was performed. Furthermore, a zero standard and a value of non-specific binding (*n*-value) were assessed and taken into account. For the *n*-value and the zero standard, the volume of the sample was replaced with 20-μL assay buffer. Furthermore, the *n*-value contained buffer instead of cortisol-glucuronide HRP complex. Every sample was pipetted in triplets, every zero standard in quadruplets and every *n*-value, as well as the standard curve values, in duplicates. For the reduction of batch effects, control group and the treated groups were always pipetted on the same ELISA plate together, for every tissue.

### Statistics and software

The different treated groups were tested for variance homogeneity with Levene’s test and on normal distribution with Shapiro–Wilk test. The tested groups were CuCl_2_-treated group (Cu_2_Cl), NaCl-treated group (NaCl), control group with a positive feed intake (CF) and control that had no feed intake (CNF). Since assumptions for a parametric test were not met, the non-parametric Kruskal–Wallis H test for multiple group comparison was applied. As the number of individuals in some cases was quite small (*n* = 10), we used Mann–Whitney U test for pairwise comparisons. We rejected the null hypothesis at the 5% significance level. For all statistical calculations, R software (R: The R Project for Statistical Computing, 2019) was used, including the following packages: car (Fox *et al.*, 2018), xlsx (Dragulescu and Arendt, 2018) and rJava (Urbanek, 2018). Statistical graphics were drawn with R (R: The R Project for Statistical Computing, 2019).

## Results

### Filtering activity

- During the experiment, there was considerable visible filtering activity in all mussels in the control group, regardless of them being provided with feed or not. In both chemical-treated groups, there was no visible filtering activity in any of the specimens, regardless of the provision of feed, most likely indicating avoidance behaviour.
- The water of all mussels in the control group, which were provided with feed, was clearly less turbid compared to all mussels of the treatment groups that were provided with feed.
- In both chemical-treated groups, none of the mussels that were provided with feed had a gastrointestinal tract filled with ingesta, but all mussels in the control group that were provided with feed had a well-filled gastrointestinal tract.

Only mussels that both filtered visibly and had a well-filled gastrointestinal tract were recorded with a positive feed intake.

### Validation of the extraction and measurement protocol

For the validation of the ELISA, pre-aliquoted cortisol–mussel extract pools without additions were used. Every value was pipetted in triplets, as it was done for every sample during the experiment. Measurement range of the cortisol was located in the range of 1.5 up to 125 pg/well/20 μL. The intra-assay and inter-assay coefficient of variation (CV) was identified for the cortisol–mussel extract. Mean intra-assay CV was 6.1%, mean inter-assay CV was 10.2%, based on an average measurement of a high and a low cortisol level (3.5 and 7.5 pg/well/20 μL) averaged over 14 plates at different days. Recovery rate for cortisol was measured via ELISA. For this purpose, mussel homogenate of one mussel pool was weighted into three glass flasks, as described under ‘cortisol extraction’ (2.1). Each of the three glass flasks contained the same amount of homogenate. Then, 500 or 1000 pg of cortisol were added into two of the flasks, and the extraction protocol was performed. A 500 pg, a 1000 pg and a non-spiked sample were always prepared at the same time. After extraction, the ELISA protocol was performed. All three samples were pipetted on the same ELISA plate in triplets to eliminate inter-assay mistakes. The procedure was repeated nine times. For the determination of the recovery rate, the non-spiked sample was subtracted from the 500 and 1000-pg samples, and then the recovery rate was calculated in percent recovery from 500 and 100 pg cortisol, together. It averaged 84.3 ± 13.2% (mean ± SD).

### Cortisol measurement in different tissues and treatments

Overall, highly significant (Kruskal–Wallis H: χ^2^ (19) = 166.91, *P* = 2.2e^−16^) differences between the levels of cortisol in tissues of the mussels were observed (*n* = 60) for CuCl_2_-treated group (Cu2Cl; *n* = 20), NaCl-treated group (NaCl; *n* = 20), control group with a positive feed intake (CF; *n* = 10) and control that had no feed intake (CNF; *n* = 10). As no filtering activity in the chemical-stressed animals was noted, mussels that had feed provided and mussels that had no feed provided were handled as one group for the treatment NaCl and Cu (II) chloride, as also described in experimental setup. In the CNF group, the highest amount of cortisol was found in the hepatopancreas, in descending order followed by the gonads and the gills and the foot and the mantle (Table 3).

The variations in the cortisol levels (pg cortisol per gramme tissue) between different tissues were most pronounced in the non-treated group (CNF). If the mussels were treated, the differences converged slightly. The Kruskal–Wallis H test showed the following significances when comparing different tissues: CNF, χ^2^ (4) = 29.051, *P* = 7.632e^−06^; CF, χ^2^ (4) = 26.055, *P* = 3.085e^−05^; CuCl_2_-treated group, χ^2^ (4) = 18.764, *P* = 0.0008743; and NaCl-treated group, χ^2^ (4) = 19.658, *P* = 0.0005834.

**Figure 1:**
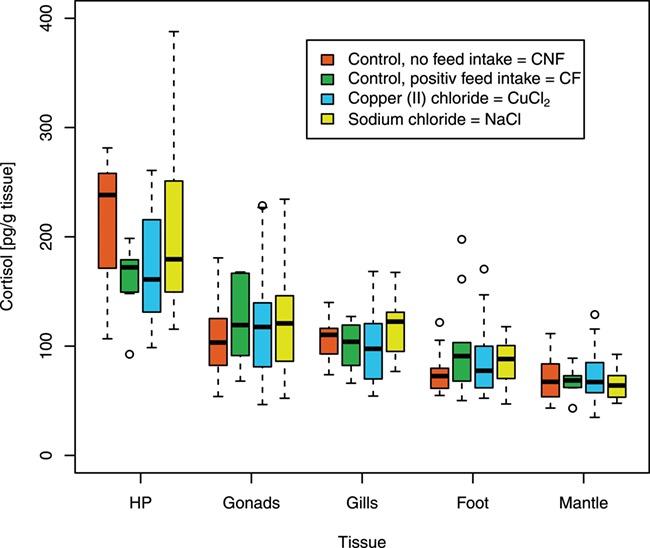
Cortisol levels in different tissues (pg cortisol per gramme tissue): the level of cortisol in five different tissues of *A. anatina* after distress treatments, feeding and in the control group (hepatopancreas, gonads, gills, foot, mantle); HP = hepatopancreas; CNF, *n* = 10; CF, *n* = 10; copper (II) chloride, *n* = 20; sodium chloride, *n* = 20; circles represent outliers, they differ more than 1.5 times from upper and lower quartile; no outliers were rejected for calculations.

**Figure 2:**
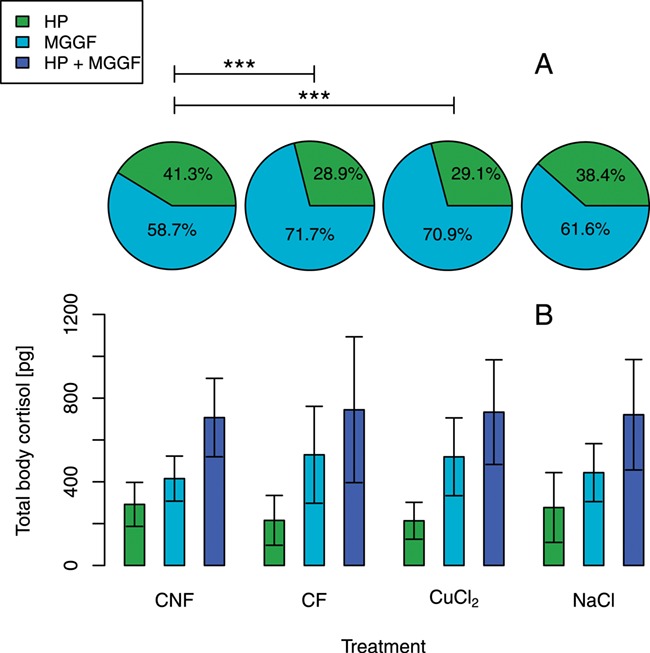
Total body cortisol—comparison of HP proportion of total body cortisol and MGGF proportion of total body cortisol; HP = hepatopancreas; MGGF = sum of mantle, gills, gonads, foot; total cortisol = hepatopancreas, mantle, gills, gonads, foot; CuCl_2_ = copper (II) chloride treatment; NaCl = sodium chloride treatment; A: pie charts are showing the distribution of total body cortisol between HP and MGGF. B: Bars are showing mean ± standard deviation of total body cortisol per mussel; *** are showing highly significant increase in the difference between HP and MGGF; CNF, *n* = 10; CF, *n* = 10; copper (II) chloride, *n* = 20; sodium chloride, *n* = 20

In the gonads and in the foot muscle, there was a very slight and non-significant, increase in the cortisol level (pg cortisol per gramme tissue) from the treated groups (CF (*n* = 10); both chemical stressor groups (*n* = 20 each)) to the non-treated group (CNF (*n* = 10)) (Fig. 1). In the gills, only NaCl-treated mussels (*n* = 20) had a slightly higher, but not significant, level of cortisol compared to CNF (*n* = 10). In the mantle, there was no difference in the cortisol level (pg cortisol per gramme tissue) between the groups. In contrast to this, a decrease of the cortisol level (pg cortisol per gramme tissue) in the hepatopancreas from CNF (*n* = 10) to the treated groups (CF (*n* = 10); both chemical stressor groups (*n* = 40)) was found (Fig. 1). Here, the Mann–Whitney U test showed a significant difference between the CNF (*n* = 10) and the CuCl_2_-treated group (*P* = 0.05, *n* = 20). Between the mussels in CNF (*n* = 10) and the mussels in CF (*n* = 10), there was no significance but still a strong trend noticeable (*P* = 0.06). Furthermore, we set a balancing (Fig. 2) between the hepatopancreas and the rest of the measured tissues (sum of mantle, gills, gonads, foot (= MGGF)). For the balancing, the cortisol level of every individual was identified for every single tissue (weight multiplied with cortisol level (pg cortisol per gramme tissue)). For MGGF, the mantle, gills, gonads and the foot were totalized, and for the total cortisol, MGGF and HP were summarized. Since the cortisol level of the heart and kidney was not measured, we had to omit them in the balancing. Due to their low weight (Table 1), and since similar cortisol levels as in MGGF can be expected, their impact can be considered as negligible. The difference in the mean total tissue cortisol, as a proportion of mean total body cortisol, between HP and MGGF was highly significant (Fig. 2A) between CNF and CuCl_2_ treatment (*P* = 0.0004), as well as when comparing CNF to CF (*P* = 0.0007). That is, we compared (MGGF [CNF] - HP [CNF]) vs. (MGGF [treatment] - HP [treatment]). There was no significant difference between CNF and the NaCl treatment.

**Table 1:** Mean tissue and whole mussel wet weight (*W_T_*) of all 60 test animals.

Tissue	Mantle	Gills	Foot	HP^a^	Gonads	^*^Kidney	^*^Heart	^*^Shell	^*^ ^b^Whole mussel
Mean *W_T_* (g)	1.85 ± 0.55	1.79 ± 1.16	0.74 ± 0.23	1.29 ± 0.40	0.92 ± 0.37	0.26 ± 0.11	0.20 ± 0.10	10.00 ± 3.34	40.32 ± 11.38

Mean ± SD.

^a^Hepatopancreas.

^*^Only weighted, no further analysis.

^b^Whole mussel wet weight was taken before dissection.

## Discussion

There is still a lack of knowledge regarding diverse physiological response patterns to stress in freshwater mussels (Fritts *et al*., 2015). Newly established methods can close this gap. In this study, we successfully established and described a practicable ELISA method for a valid and sensitive measuring of cortisol levels from different body tissues. The recovery rate of cortisol and the reproducibility of the testing system both suggest that this methodology is suitable for routine testing. The conversion of the formerly customary di-ethyl ether-based extraction method to ethyl acetate and the used ether mixture make the technical realization of the method more practicable and safer. Different tissues strongly varied in their cortisol concentration. The by far highest level of cortisol was detected in the hepatopancreas followed by the gonads. The high level of cortisol in the hepatopancreas suggests that cortisol is either accumulated or even produced and stored in this tissue. In case of environment-borne cortisol accumulation in the mussels, we would not expect that environmental influences could cause redistribution among tissues. In our experiment, we noticed a treatment-specific distribution of cortisol, comparing different tissues. Cortisol levels (pg per gramme body weight) significantly decreased in the hepatopancreas when the mussels were stressed with CuCl_2_ as well as in CF, compared to CNF. This could be due to a redistribution of cortisol from the hepatopancreas to other tissues. We did not observe significant increases in the other tissues comparing treatment groups and the control group (pg cortisol per gramme tissue), but even a slight increase in all tissues could quite possibly explain a significant decrease in the hepatopancreas. To assess if such a slight increase in MGGF was present, based on a redistribution of cortisol from hepatopancreas to other tissues, we balanced total body cortisol (Fig. 2). If cortisol would be produced or would be stored in the mussels purposefully, the hepatopancreas, as a gland tissue, could be the tissue where those processes take place. The results demonstrate that in CF and during CuCl_2_ treatment, the cortisol in HP comprised less than 30% of total body cortisol compared to CNF group where it comprised more than 40%. Therefore, a redistribution of cortisol from hepatopancreas to the other tissues is highly likely in these two cases. There was no significant increase in the cortisol of all tissues together (MGGF + HP, Fig. 2B) in the treated groups, compared to CNF. This indicates that there was no or only a minor additional cortisol production or accumulation in the treated mussels at this point in time (i.e. 24 hours after start of treatment). No redistribution of total body cortisol between HP and MGGF was evident, when comparing the NaCl treatment and CNF. There are diverse possibilities why the contact of the mussels to a stressor like NaCl did not cause a redistribution of total body cortisol from one tissue to the other. On the one hand, it is possible that NaCl in the experimental setup (*inter alia*, the seasonal low temperature) was not as fast-acting as a toxin compared to CuCl_2_. On the other hand, it could also be that the mussels experienced no stress because of a fast recognition of the stressor and their evident response by shell closure. None of the chemical-treated mussels showed filtration during the treatments, even when we provided feed (Table 2). With this known avoidance strategy (Hartmann *et al.*, 2016), it would be possible that the animals were able to avoid direct tissue exposure to the stressor NaCl. The increased redistribution of cortisol from the hepatopancreas to other tissues is indicating that mussels were not able to escape the CuCl_2_ treatment completely, despite shell closure.

**Table 2:** Filtering activity of control group and both treatments.

Group	Control group (*n* = 20)		Sodium chloride (*n* = 20)		Copper (II) chloride (*n* = 20)	
Feed intake	Yes	No	Yes	No	Yes	No
Number	10	10	0	20	0	20

**Table 3:** Cortisol in different tissues: mean cortisol (in pg) per gramme tissue in the control group that has no feed intake (CNF, *n* = 10).

Tissue	HP^a^	Gonades	Gills	Foot	Mantle
Pg cortisol per gramme tissue	213.82 ± 57.96	105.48 ± 36.56	106.53 ± 19.58	76.95 ± 21.30	70.40 ± 22.47

Mean ± SD.

^a^Hepatopancreas.

## Conclusion

Our study demonstrated that there are changes in the biodistribution of the steroid hormone cortisol in freshwater mussels after exposure to external stressors. As we were able to show this change in biodistribution, it can be hypothesized that cortisol is not accidentally stored in the hepatopancreas only due to its affinity to fatty tissue. An explicit link to a distressor cannot be made directly, because change in biodistribution was equally strong in the distressor CuCl_2_ treatment as in the CF, compared to CNF. Therefore, it has to be assumed that an eustressor or even the stimulation of the metabolism can cause cortisol redistribution similar to a distressor. Nevertheless, further studies with this corticosteroid in mussels could eventually help to assess the condition of mussel populations, which would improve their value as bioindicators for the monitoring of aquatic ecosystems. As evident from this study, different tissues should be included when measuring cortisol in mussels, because of the possibility of an inverse reaction of the hepatopancreas compared to other tissues. Measurements of whole body extracts are not recommended since there is a bias due to tissue-specific redistribution. During the first 24 hours of stressor exposure, the mussels did not seem to react with an increase in total body cortisol and mostly with a redistribution of cortisol. With the knowledge that an eustressor or metabolism stimulation can cause quite similar reactions in the mussels like a distressor, the measurement of cortisol in mussels for stress assessment has to be planned and interpreted diligently. It should be ensured that mussels cannot avoid a stressor regime, for example, by choosing a difficult-to-avoid stressor or by choosing a suitable duration of exposure. Furthermore, we have to point out that cortisol levels can be species and life-stage specific, and results from *A. anatina* have to be extrapolated to other mussel species with care. It can be hypothesized, though, that the principle of biodistribution of cortisol in most mussel species is quite similar to *A. anatina*. Since there is little information available on the regulation of cortisol in freshwater mussels and since the physiological reason for the presence of steroid hormones in invertebrates is still discussed controversially (Lafont and Mathieu, 2007; Fernandes *et al.*, 2011), further experiments with the described detection method could help in understanding the fate and function of cortisol in invertebrates and particularly in mussels.

## Supplementary Material

supplementary_coz101
