# Supplementary material for: Does environmental stress affect cortisol biodistribution in freshwater mussels?
Source: Conserv Physiol. 2019 Dec 8;7(1):coz101. doi: 10.1093/conphys/coz101 (PMC6899224; doi:10.1093/conphys/coz101)
Supplement: supplementary_coz101 [file supplementary_coz101.docx]

**Supporting information**

**Table S1. Water chemistry parameters**

| *Parameter* | *Concentration* |
| --- | --- |
| Sodium (Na+) | 27 mg L-1 |
| Potassium (K+) | 3.3 mg L-1 |
| Calcium (Ca2+) | 91.7 mg L-1 |
| Magnesium (Mg2+) | 26.4 mg L-1 |
| Iron (Fe3+) | <0.01 mg L-1 |
| Manganese (Mn2+) | <0.01 mg L-1 |
| Chloride (Cl-) | 38 mg L-1 |
| Total Hardness | 3.4 mmol.-1 CaCO3 |

Water chemistry parameters, measured during the acclimatization and during the experiment for the control group. In the sodium chloride and copper(II) chloride treated groups, the same water chemistry parameters were measured, except for sodium and chloride parameters (see 2.2 “Experimental setup”).

**ELISA buffer**

washing buffer: 0.5 g/L Tween 80 (Merck, Germany)

reaction substrate A: 1 g/L Hydrogen peroxide urea (CH₄N₂O * H₂O₂, Merck, Germany), 18 g/L disodium hydrogen phosphate dihydrate (Na2HPO4 * 2H2O, AppliChem, Germany),10.3 g/L citric acid 1-hydrate (AppliChem, Germany)

reaction substrate B: 500 mg Tetramethylbenzidine (TMB, Sigma, Germany) solved in 40 ml dimethyl sulfoxide (DMSO, Sigma, Germany), 10.3 g citric acid 1-hydrate (AppliChem, Germany) solved in 960 ml distilled water

assay buffer: 7.12 g/L disodium hydrogen phosphate dihydrate (Na2HPO4 * 2H2O, AppliChem, Germany), 8.5 g/L sodium chloride (AppliChem, Germany), pH 7.2 (calibrated with 1M HCL (AppliChem, Germany))
